# Comparison of outcomes between early and late tracheostomy for critically ill patients

**DOI:** 10.1186/cc12100

**Published:** 2013-03-19

**Authors:** K Suzuki, S Kusunoki, T Yamanoue, K Tanigawa

**Affiliations:** 1 Hiroshima Prefectural Hospital, Hiroshima, Japan; 2 Hiroshima University Hospital, Hiroshima, Japan

## Introduction

Tracheostomy is one of the more commonly performed procedures in critically ill patients requiring long-term mechanical ventilation. However, the optimal timing or method of performing tracheostomies in this population remains to be established. In the present study, we compared outcomes of early and late tracheostomy in critically adult patients with different clinical conditions.

## Methods

All patients needing tracheostomy in the Critical Care Medical Center of Hiroshima Prefectural Hospital from January 2009 to December 2011 were surveyed. Patients with tracheostomy who were not indicated for mechanical ventilation were excluded from the subjects. Early tracheostomy (ET) was defined as <10 days after tracheal intubation and late tracheostomy (LT) was defined as ≥10 days after intubation. We compared patient characteristics, type of tracheostomy procedure, length of weaning from ventilator and outcomes between the groups. Data are shown as the mean ± SD, with unpaired *t *test and Mann-Whitney U test used for statistical analyses. Statistical significance was accepted at *P <*0.05.

## Results

One hundred patients were surveyed. The ET and LT groups included 49 and 51 patients, respectively. Tracheostomy was performed using a percutaneous procedure in 48 patients (ET: 25, LT: 23) and a surgical procedure in 52 patients (ET: 24, LT: 28). Sixty-two patients (ET: 34, LT: 28) survived to discharge and 16 patients died in the ICU (ET: 7, LT: 9). Fifty-six patients (ET: 31, LT: 25) were weaned from ventilator support and tracheostomy cannula was removed in 20 patients (ET: 11, LT: 9). There were no significant differences in type of tracheostomy procedure, period from tracheostomy until ICU and hospital discharge, rate of patients who could be weaned from ventilator and removed tracheostomy cannula, and ICU and hospital mortality between the groups. The length of mechanical ventilation and the time to removal of tracheostomy cannula were significantly shorter in the ET group (5 ± 7 vs. 26 ± 41 and 29 ± 24 vs. 94 ± 83 days, respectively).

## Conclusion

In this retrospective study, early tracheostomy reduced the length of weaning after tracheostomy and the time to removal of tracheostomy cannula, while there were no differences in the length of ICU stay and patient outcome. In critically ill adult patients who require mechanical ventilation, a tracheostomy performed at an earlier stage may shorten the duration of artificial ventilation. A further randomized clinical trial is essential to determine the effectiveness and safety of early tracheostomy.
